# Steviol Glycosides (E960) From *Stevia rebaudiana*: Technological Innovations, Safety Assessment, Regulatory Aspects and Their Role in Sustainable Food Systems

**DOI:** 10.1002/fsn3.71891

**Published:** 2026-06-29

**Authors:** Aswin Sakthivel Manoharan, Ramesh Kumar Selvan

**Affiliations:** ^1^ Department of Horticulture and Food Science VIT School of Agricultural Innovations and Advanced Learning (VAIAL), Vellore Institute of Technology Vellore Tamil Nadu India

**Keywords:** natural sweetener, rebaudioside A, *Stevia rebaudiana*, steviol glycosides, stevioside

## Abstract

Stevia ( *Stevia rebaudiana* Bertoni) is a small perennial herb widely recognized as a natural sweetener. Its leaves contain zero‐calorie, non‐nutritive, and high‐potency sweetening compounds with promising pharmaceutical potential, particularly for diabetes management. Steviol glycosides in *Stevia rebaudiana* leaves exhibit therapeutic potential against Type 2 Diabetes Mellitus (T2DM). Stevioside and rebaudioside A enhance insulin secretion and improve insulin sensitivity, helping regulate blood glucose levels. Steviol glycosides, designated as E960, are approved food additives in the European Union and permitted for use across various food categories under specific manufacturing and quality standards. Their application falls under the rules set by the European Parliament and Council Regulation (EC) No. 1333/2008, which governs the use of food additives. The European Union revised its labelling regulation, classifying E960 as “steviol glycosides from Stevia” (E960a) to highlight its natural plant origin. However, the global regulatory framework and acceptable daily intake (ADI) for steviol glycosides remain inadequately standardized. This review discusses Stevia and Steviol Glycosides as a sustainable source of non‐nutritive sweeteners, advances in extraction, safety assessment, toxicological evaluation, global regulatory framework, acceptable daily intake (ADI), stevioside traceability, applications in functional foods and health‐oriented product development, consumer acceptance, labelling trends and prospects of Steviol Glycosides.

## Graphical abstract

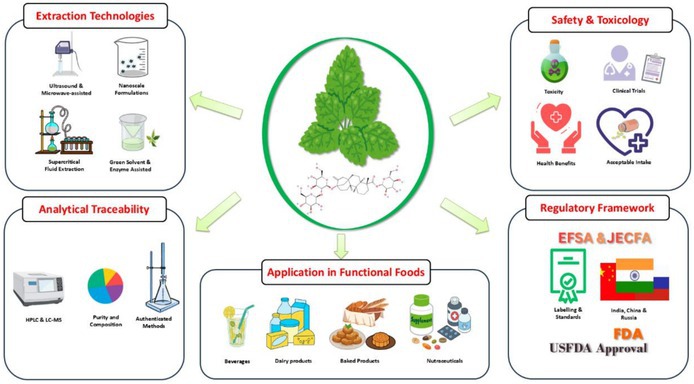

## 1 Introduction

Sweetness is a highly preferred sensory attribute in foods and plays an important role in consumer acceptance. However, excessive consumption of sucrose, the primary dietary source of sweetness, is associated with several metabolic disorders, leading to growing interest in alternatives. Consequently, the food industry increasingly utilizes artificial and non‐nutritive sweeteners as low‐calorie substitutes to provide sweetness while reducing sugar intake (Raghavan et al. 2023; Singh and Rao 2005). Non‐nutritive Sweeteners (NNS) refer to food additives used to add sweetness to food substances or as table‐top sweeteners. Such compounds have extensive applications as sugars in food, beverage and pharmaceutical products because of their low caloric content and high sweetness, contrasting with sugar (Fitch et al. 2021). *Stevia rebaudiana* , a plant native to South America, has gained considerable attention as an environmentally sustainable alternative to artificial sweeteners. This is primarily attributed to its intense natural sweetness and plant‐derived origin, along with its relatively lower environmental footprint compared with conventional sugar‐producing crops such as sugar beet and date palm (Ciriminna et al. 2019; Prakash et al. 2014). Stevia is widely considered an ecologically friendly alternative to conventional sugars because its high sweetness potency, and significantly lower greenhouse gas emissions and land requirements allow equivalent sweetness with substantially reduced environmental impact (Ashwell 2015). Stevioside compounds have been determined by regulatory bodies such as the U.S. Food and Drug Administration (FDA), European Food Safety Authority (EFSA) and the Joint FAO/WHO Expert Committee on Food Additives (JECFA) as generally recognized as safe (GRAS) when used as per the accepted acceptable daily intake (ADI) levels (Kamanzi 2025; Lohner et al. 2020). Steviol glycosides are widely used in diverse food categories. Besides, the recent research highlighted their possible functional advantages, including anti‐inflammatory, anti‐obesity, antidiabetic, anti‐hypertensive, anti‐caries and anti‐cancer effects (Huang et al. 2024), making them a promising multifunctional ingredient for the development of functional food products. The nanosizing of stevia extract to improve absorption and promote better therapeutic performance, and the production of stevia fortified capsules for a daily alternative to sugars, are also commercially produced. The European stevia market was estimated at US$150.8 million in the year 2021, and it is set to increase to US$233.63 million in the year 2026 with a compound annual growth rate (CAGR) of 9.15.

**FIGURE 1:**
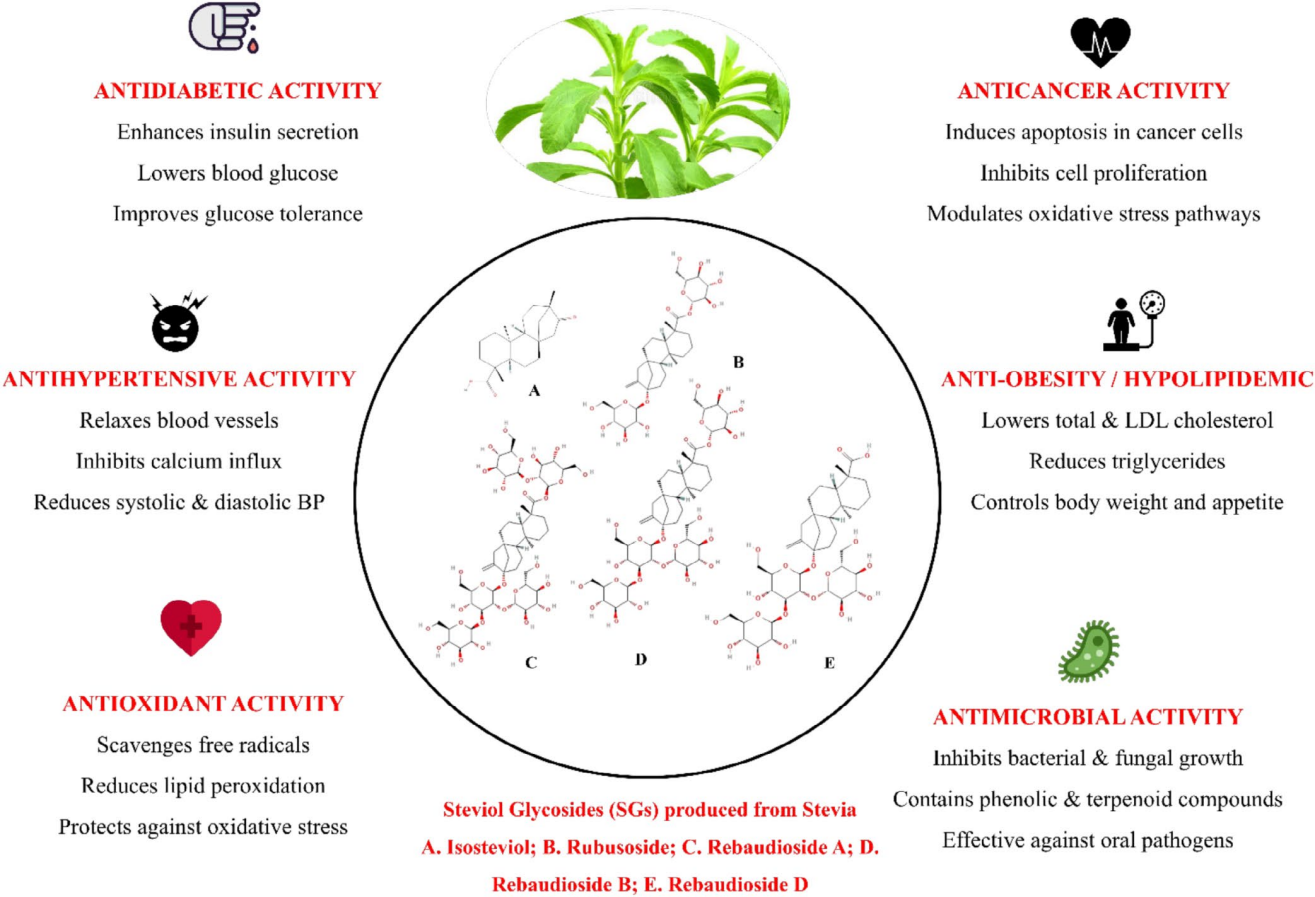
Pharmacological and therapeutic activities of steviol glycosides (Peteliuk et al. 2021; Wang et al. 2025).

In the year 2025, China became the number one exporter of stevia extract to Europe under HS code 29389090. China produces 80% of the stevia in the world. India ranked as the third‐largest supplier of stevia extract to the European Union among non‐EU exporting countries. The stevia industry in the country is slowly growing with the support of several government programs (Exposure Assessment EFSA 2011). To illustrate, the National Bank of Agriculture and Rural Development (NABARD) grants, low‐interest loans, and subsidy programs aimed at supporting growers of stevia. However, Indian farmers have difficulties in embracing stevia farming because of the lack of funds and the high capital needed to farm stevia. Food additives that have been approved to be used in the European Union are designated E‐numbers, meaning that the additives have been tested sufficiently and authorized to be consumed. Stevia extract is assigned the following two codes: E 960a: steviol glycosides obtained directly by *Stevia rebaudiana* , and E 960c: steviol glycosides modified enzymatically. It may be applied as a sugar replacement in drinks, milk products, candy, and bakery products (Exposure Assessment EFSA 2011; Regulation (EU) 1169/2011 (EU 2011) 2011). A majority of the products that are sold as sweeteners are synthetic substances such as saccharin, aspartame, and acesulfame‐K. Nevertheless, there is a growing alarm over their possible health effects, which has prompted the regulatory bodies to come up with maximum permissible levels to mitigate the risk of toxicity. Consequently, natural sweeteners are attracting increased attention as safe and healthier alternatives to artificial sweeteners in the food industry (Puri et al. 2012). Natural sweeteners, such as steviol glycosides derived from *Stevia rebaudiana* , are often regarded as healthier options due to their origin (Ikechukwu et al. 2017). Unlike conventional sugar sources such as sugarcane and sugar beet, *Stevia rebaudiana* produces steviol glycosides, a group of natural high‐intensity sweeteners widely used as Non‐nutritive sweeteners (NNS) (Chatsudthipong and Muanprasat 2009; Lemus‐Mondaca et al. 2012). Replacing sugars with artificial and natural sweeteners is a globally accepted measure to fight obesity, hyperlipidemia, diabetes, and cavities (Ahmad and Ahmad 2018a). Steviol glycosides can now be produced commercially through microbial fermentation using engineered microorganisms such as *Saccharomyces cerevisiae* or *Yarrowia lipolytica*, enabling sustainable large‐scale production of high‐purity compounds like rebaudioside M (Luu and Atsumi 2025). Stevioside has been applied in the treatment of disrupted carbohydrate metabolism, including diabetes, obesity, and hypertension, but its use is limited by the unpleasant odor and bitter astringent taste of some glycosides and alkaloids in the extract. On the contrary, rebaudioside A has a sweet, non‐bitter taste (Carakostas et al. 2008). The pharmacological and therapeutic activities of Steviol Glycosides are shown in Figure 1.

The scope of this review is to provide a comprehensive overview of steviol glycosides derived from *Stevia rebaudiana* , focusing on recent advances in extraction technologies, analytical traceability, safety assessment, regulatory frameworks, and applications in functional foods. The objective is to synthesize current knowledge and highlight key research gaps to support the sustainable use of steviol glycosides in modern food systems. Although numerous studies have investigated steviol glycosides from *Stevia rebaudiana* , most existing reviews focus on specific aspects such as extraction methods or biological activities, with limited integration of technological, safety, regulatory, and application perspectives. Challenges related to analytical traceability, standardization of regulatory frameworks, and large‐scale sustainable production remain inadequately addressed. Therefore, this review provides a comprehensive synthesis of recent advances in extraction technologies, safety evaluation, regulatory status, and functional food applications of steviol glycosides, while highlighting key research gaps and future prospects.

## 2 Research Methodology

**FIGURE 2:**
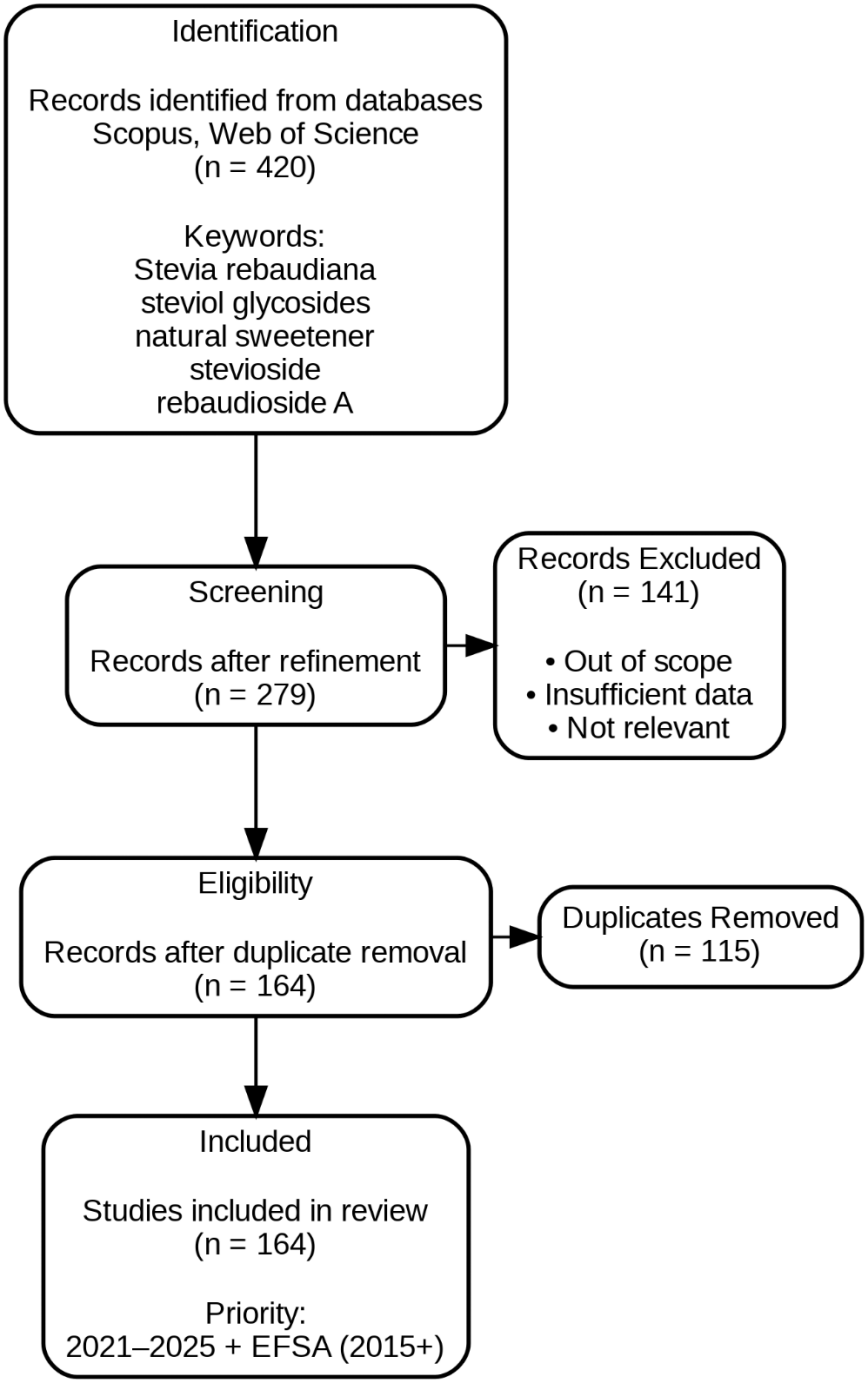
Flow diagram of study identification, screening, eligibility, and inclusion.

A comprehensive review of the available literature was carried out to collect relevant scientific publications on the Steviol Glycosides, advances in extraction, safety assessment, toxicological evaluation, global regulatory framework, acceptable daily intake (ADI), applications in functional foods and health‐oriented product development, consumer acceptance, and labelling of stevia. A literature survey was done from major platforms, including Scopus, Google Scholar, ScienceDirect, Web of Science, and similar databases covering publications up to October 2025. The search was directed using keywords such as *Stevia rebaudiana* , steviol glycosides, natural sweetener, stevioside, and rebaudioside A. Other studies that were beyond the scope, whose details were not sufficient, or whose relevance was not met were eliminated. Relevance and relatedness were considered in the selection process so that only the most significant and suitable information could be included. The literature search was conducted using the article title, abstract, and keywords in the Scopus and Web of Science (WoS) databases, initially identifying approximately 420 publications. After refining and adjusting the search parameters, 279 articles were retained for further consideration. The records retrieved from both databases were subsequently merged and exported for additional screening. During the data cleaning process, duplicate entries were identified and removed, resulting in a final dataset comprising 164 unique publications related to steviol glycosides. Priority was given to articles published within the last 5 years (2021–2025). However, a limited number of essential studies from the previous decade were also included where necessary, along with key publications from the European Food Safety Authority (EFSA) published from 2015 to provide important regulatory context. The flow diagram of Study Identification, Screening, Eligibility, and Inclusion is included in Figure 2.

## 3 *Stevia rebaudiana* As a Sustainable Source of Non‐Nutritive Sweeteners

### 3.1 Dietary Sugar, Artificial Sweeteners and Associated Health Risks

The sweet taste of *Stevia rebaudiana* leaves is mainly due to diterpene glycosides, principally stevioside and rebaudioside A, which are approximately 200–300 times sweeter than sucrose (Lemus‐Mondaca et al. 2012). Sucrose provides empty calories (4 kcal g^−1^) and lacks essential nutrients. Regular consumption of sugar‐sweetened beverages (SSBs) further increases calorie intake and glycaemic load. Non‐nutritive sweeteners (NNSs), including steviol glycosides, are sweeter than sugar and contain very low or no calories, helping reduce daily sugar and energy intake and supporting weight management (Muñoz‐Labrador et al. 2024). Sucrose is one of the most widely consumed dietary sugars worldwide and is commonly used as a sweetener in processed foods and beverages, making it a major contributor to daily carbohydrate intake in modern diets (Macdonald 2016). Dietary sucrose produces a rapid postprandial increase in blood glucose and insulin secretion, contributing to higher glycaemic responses compared with complex carbohydrates. Chronic high intake of sucrose and other added sugars has been linked to increased body weight, impaired insulin sensitivity and higher risk of metabolic diseases such as type 2 diabetes and cardiovascular disorders (Veit et al. 2022).

A large prospective cohort study involving 103,388 adults from the NutriNet‐Santé study investigated the association between artificial sweetener intake and cardiovascular disease. Using repeated 24‐h dietary records and Cox hazard models, researchers found that higher consumption of sweeteners such as aspartame and acesulfame‐K was associated with increased cardiovascular and cerebrovascular disease risk (Debras et al. 2022; Hong et al. 2025). Numerous studies indicate that artificial sweeteners may adversely affect human health by altering gut microbial composition, influencing blood glucose regulation, and impacting cardiovascular function. For instance, a recent study reported that erythritol consumption may promote thrombosis and elevate the risk of stroke and myocardial infarction (Debras et al. 2022; Witkowski et al. 2023). Research conducted in Israel demonstrated that artificial sweeteners can modify glucose metabolism in humans, potentially causing disturbances in normal blood glucose regulation (Suez et al. 2022). Aspartame consumption has been shown to alter gut microbiota composition by increasing the abundance of Enterobacteriaceae while reducing beneficial bacteria such as *Clostridium* cluster XI, a probiotic group. The decline of these beneficial microbes may facilitate the proliferation of pathogenic bacteria and the production of harmful metabolites (Feng et al. 2024). A long‐term prospective cohort study involving 61,440 women over 18 years reported that frequent consumption of artificial sweeteners in tablet or sachet form was associated with a higher incidence of type 2 diabetes (Debras et al. 2022; Fagherazzi et al. 2017). Artificial sweetener intake may stimulate overeating behavior and reduce the secretion of metabolic hormones such as GLP‐1, which can impair glucose homeostasis and potentially increase the risk of diabetes development (Fagherazzi et al. 2017).

### 3.2 Botanical and Compositional Characteristics of *Stevia rebaudiana*

Stevia leaves are rich in nutrients, including proteins, lipids, essential amino acids, carbohydrates, vitamins, and minerals. The nutritional composition of stevia can vary depending on cultivation practices and environmental conditions (Clemente et al. 2021). Today, sixty‐four steviol glycosides (SGs) and more than thirty sweet‐tasting compounds in Stevia leaves are known (Aswin Sakthivel and Ramesh Kumar 2025). Dried Stevia leaves have 11.2 to 16.0 g of protein, 61.9 g of carbs, 1.9 to 3.73 g of lipids, and 6.8 to 15.2 g of bulk per 100 g of dried mass (Khiraoui et al. 2017). The presence of several phenolic compounds with potent antioxidant capabilities was discovered in Stevia leaves. The methanolic extracts contained total polyphenols and flavonoids at concentrations of 25.18 and 21.73 mg g^−1^, respectively. Stevia leaves are also rich in water‐soluble vitamins, including vitamin C (14.98 mg 100 g^−1^), riboflavin (0.43 mg 100 g^−1^), and folate (52.18 mg 100 g^−1^) (Kim et al. 2011). Rebaudioside A exhibits the superior flavor profile, whereas stevioside is mainly responsible for the bitter aftertaste of stevia, often described as a “liquorice‐like” taste (Lemus‐Mondaca et al. 2012). A wide range of SGs is found in Stevia leaves, each occurring in different proportions (Ciriminna et al. 2019). The sweetness equivalence of Rebaudioside A extract containing 60% purity is typically 200 to 300 times that of sugar. Steviol glycosides (SGs) have a very high sweetening capacity, 450 times that of sucrose. These compounds are highly utilized as sweeteners, flavor and sugar substitutes due to their thermal stability. Besides, they do not influence glycemic index and do not lead to an increase in energy storage in the body (Gençdağ et al. 2021; Kurek et al. 2020).

Rebaudioside M, although present in Stevia leaves at very low concentrations (< 1%), exhibits superior sensory qualities when compared to the predominant glycosides, stevioside and Reb A, and provides a sweetness profile that closely mimics that of sucrose (Younes et al. 2019). Regulation (EU No 231 (2012)) defines purity and contamination of steviol glycosides and sets different purity requirements for those produced by the use of an enzyme. This is necessary due to the possibility of impurities produced during the enzymatic synthesis of rebaudioside M that are not similar to those produced when water is used to extract steviol glycosides (E 960a) followed by recrystallization. Purity standards for steviol glycosides derived from *Stevia* (E 960a) and for those produced through enzymatic processes, E 960c, are shown in Table 1.

**TABLE 1:** Purity requirements for steviol glycosides from stevia, E 960a and enzymatically produced steviol glycosides, E 960c.

E960a		E960c	
Total ash	Under 1%	Total ash	Under 1%
Drying Loss	< 6% (105°C, for 2 h)	Drying Loss	< 6% (105°C, for 2 h)
Residual solvents	< 200 mg/kg methanol	Residual solvents	< 5000 mg/kg ethanol
	< 5000 mg/kg ethanol		
Arsenic	< 1 mg/kg	Arsenic	< 0.015 mg/kg
Lead	< 1 mg/kg	Lead	< 0.2 mg/kg
		Cadmium	< 0.015 mg/kg
		Mercury	< 0.07 mg/kg
		Residual protein	< 5 mg/kg
		Particle size	< 74 μm (using a mesh 200 sieve and particle size < 74 μm)

*Source:* Regulation (EU) No 231 (2012).

### 3.3 Chemical Structure and Classification of Steviol Glycosides

The most predominant SG components are Stevioside (4%–13%), Rebaudioside A (2%–4%), Rebaudioside C (1%–2%), Dulcoside A (0.4%–0.7%), and Rebaudioside D/M (< 0.5%) (Gasmalla et al. 2017; Li et al. 2021). Stevia produces several glycosides that originate from steviol (13‐hydroxy‐ent‐kaur‐16‐en‐19‐oic acid) (Aswin Sakthivel and Ramesh Kumar 2025), a diterpenoid compound predominantly present in its leaves (Lemus‐Mondaca et al. 2012). The flavor characteristics of the glucosyl family of steviol glycosides (SGs) show that an equal number of C19‐linked sugar groups is positively correlated with sweetness as the number of C13‐linked groups increases, while bitterness decreases. An equal number of C13‐linked sugars, however, can decrease both sweet and bitter tastes, depending on how many C19‐linked sugar units are present (Fayaz et al. 2018). Additional glucose units are used to make glucosylated steviol glycosides by the addition of 1 to 20 molecules of glucose to steviol glycosides obtained using the *Stevia rebaudiana* leaf extract. This reaction utilizes enzymes to transfer glucose molecules from a starch source to the SGs, forming a mixture of approximately 80%–92% glucosylated steviol glycosides and 5%–15% parent compounds. The glucosylated derivatives are about 167 times sweeter than sucrose (Younes et al. 2018). Lipids found in the leaves of Stevia are considered to be rich in monounsaturated fatty acids, including oleic acid, polyunsaturated fatty acids, linoleic acid and alpha‐linolenic acid. These fatty acids have the potential to help lower blood cholesterol levels (Abbas Momtazi‐Borojeni et al. 2017).

### 3.4 Sustainability and Environmental Impact

The increasing use of steviol glycosides in food, beverages, and pharmaceuticals significantly contributes to the market's sustained expansion. Increasing awareness of natural sweeteners over synthetic options is crucial to promoting health‐conscious diets, leading to a rise in demand for steviol glycosides, naturally derived compounds from the stevia plant. The global steviol glycoside market was valued at USD 5312.6 million in 2024 and is projected to reach USD 7150.6 million by 2030, registering a CAGR of 5.2% during 2025–2030, driven primarily by increasing consumer preference for natural, plant‐based sweeteners in response to rising health and wellness awareness (Grand View Research 2024). Regionally, North America dominated the market with a 35.4% revenue share in 2024, supported by growing concerns over sugar consumption in the United States, while Europe is expected to emerge as the fastest‐growing region. In terms of product segmentation, stevioside accounted for the largest share (41.2%) in 2024, and by end use, the food sector led the market with a 46.1% revenue contribution (Grand View Research 2024; Stevia Market 2026).

Life cycle assessment (LCA) studies of steviol glycosides reveal that their production results in a much lower global warming potential and reduced land use compared to conventional sugar, positioning them as an environmentally sustainable alternative sweetener (Ahmad et al. 2020). In terms of environmental impact, the global warming potential of steviol glycosides is about 20.25 kg CO_2_‐eq per kilogram, which represents only about 5.7% to 10.2% of the carbon footprint associated with conventional sugar (Suckling et al. 2023). The land use implication is also minimized as stevia consumes 5.6%–7.2% of the land area required to produce sugar (Suckling et al. 2023). Egypt has been experiencing a marked reduction in sugar availability since 2023, primarily due to a worsening water shortage that has limited national sugar production. Egypt is characterized by arid climatic conditions and significant water scarcity, relying predominantly on the Nile River for its freshwater supply. Under these constraints, water‐intensive crops such as sugarcane and sugar beet present sustainability challenges, highlighting the potential of low‐water‐demand crops such as *Stevia rebaudiana* as alternative sweetener sources. This challenge has intensified the need for viable substitutes to conventional sugar crops. Given the country's heavy sugar import burden and restricted freshwater supply, *Stevia rebaudiana* stands out as a strategic option to replace sugarcane and sugar beet. Conventional crops demand much more irrigation. Sugarcane typically uses 10,000–12,000 m^3^/ha, and sugar beet about 6000–7000 m^3^/ha, whereas stevia cultivation requires only around 3000–4000 m^3^/ha (Abouelela et al. 2025; Hamed et al. 2021).

### 3.5 Bioactivity and Potential Health Effects

Stevia is a safe and non‐toxic natural substitute for artificial sweeteners (ASs). It is widely incorporated into various foods, beverages, and dietary supplements as a healthier alternative to synthetic sweetening agents (Khan 2015). Stevioside, derived from *Stevia rebaudiana*, exhibits strong antihyperglycemic activity, enhances insulin release, and suppresses glucagon in type 2 diabetic rats, underscoring its promise as a potential antidiabetic compound (Jeppesen et al. 2002). Studies indicate that reducing sugar consumption is most effective for weight management when accompanied by an overall decrease in total calorie intake, resulting in improved energy balance and the maintenance of a healthy body weight (Dunford et al. 2018; Te Morenga et al. 2013). In diabetic rats, administration of both Stevia and nano‐Stevia led to reductions in hyperglycemia, anxiety, and memory deficits, with nano‐Stevia showing a more pronounced effect. These findings highlight its potential for managing diabetes‐associated metabolic and neurological disorders (Khakpai et al. 2023). *Stevia* seems to exhibit a more neutral or even beneficial metabolic profile, potentially enhancing insulin sensitivity while having minimal impact on glucose absorption (Mohd‐Radzman et al. 2013). It has been demonstrated in metabolic investigations that rebaudioside M is rapidly deglycosylated by the human gut microbiota to become the major metabolite steviol, which is also analogous to the metabolism of other steviol glycosides. Such glycosidic bonds cannot be broken by the human digestive enzymes but are broken by the intestinal microflora.

Nano‐Stevia denotes a nanoscale form of Stevia in which its bioactive compounds are encapsulated or engineered at the nanometer scale to boost their stability, bioavailability, and overall efficacy. This nanosizing strategy is believed to enhance absorption and deliver superior therapeutic effects compared to standard Stevia extracts (Ahmad et al. 2023). Evidence from multiple studies on the wide‐ranging biological effects of Stevia indicates that its in vivo metabolites, including steviol, isostearyl alcohol, and various polyphenolic compounds contribute to the modulation of glucose regulation pathways (Li et al. 2021), preventing cardiovascular diseases (Olas 2022), exhibiting a broad spectrum of biological functions, including suppression of bacterial proliferation, protection against dental caries, anti‐inflammatory effects, cancer‐preventive potential, and several other important pharmacological properties. According to recent research, stevia‐derived compounds can be utilized in the treatment of cardiovascular diseases (CVD) and some tumors (Ferrazzano et al. 2015; Ilias et al. 2021). Stevioside has a massive potential for reducing the level of glucose in the blood of diabetic rat models, confirmed by a number of lines of evidence (Aswar et al. 2019; Kurek et al. 2022). Blood sugar‐lowering effect is primarily linked to its capacity to stimulate insulin secretion. The increasing global incidence of metabolic disorders, such as obesity, type 2 diabetes, and cardiovascular diseases, has prompted significant efforts to reduce dietary sugar intake (Kamanzi 2025; Lara‐Castor et al. 2025).

A 3‐month open‐label RCT replacing sugar with a stevia‐based sweetener in overweight including prediabetic and diabetic adults showed significant reductions in body weight and waist circumference, a non‐significant decrease in HbA1c, and no safety concerns (Raghavan et al. 2023). It is found that stevia significantly reduced fasting blood glucose, particularly in individuals with higher BMI or metabolic conditions, while effects on insulin and HbA1c were non‐significant and of low certainty (Zare et al. 2024). In the case of appetite, a GRADE‐assessed analysis of 11 RCTs (428 participants) found no significant overall effect of stevia, although a slight increase in “desire to eat” was observed in one subgroup, with evidence certainty ranging from very low to moderate (Zare et al. 2025). Human studies indicate that steviol glycosides do not markedly alter the core gut microbiota, although minor changes have been observed in certain taxa, such as butyrate‐producing groups similar to *Faecalibacterium* (Kasti et al. 2022). A randomized controlled trial in healthy adults found that 12 weeks of real‐life stevia consumption did not significantly affect overall gut microbiota composition or diversity; only minor shifts were observed in a few taxa, including *Butyricoccus* and *Akkermansia*, without evident clinical relevance. Stevia extracts exhibit antiglycaemic and antioxidant effects in adipose and vascular tissues, can reduce blood pressure in certain hypertensive groups, and may help stabilize atherosclerotic plaques (Peteliuk et al. 2021). A study comparing stevia‐containing sweeteners with sucrose, aspartame, and xylitol found that stevia‐based products did not contribute to dental caries and were less cariogenic than sucrose, highlighting their potential benefits for oral health (Augustinho do Nascimento et al. 2021).

## 4 Extraction of Steviol Glycosides and Its Traceability

### 4.1 Recent Advances in Extraction of Steviol Glycosides (SGs)

Steviol glycosides are usually extracted using aqueous or alcoholic solvents, with the possible assistance of an auxiliary energy source (such as liquid pressurization, ultrasounds, or microwaves), followed by clarification and purification (Ameer, Bae, Jo, Chung, et al. 2017; Das et al. 2015). Conventional methods generally rely on liquid solvents, often at elevated temperatures, which present certain drawbacks such as low extraction yield and reduced quality, due to the decomposition of compounds that affect purity. The production process consists of two major steps: the extract is first obtained as a result of water extracting the leaves of *Stevia rebaudiana* Bertoni, and then the extract is pre‐purified. These techniques also involve high processing costs because of the large amounts of solvent and energy required (Braga et al. 2018; Javad et al. 2014; Kovačević et al. 2018). The common drawbacks of standard extraction processes are that extraction is slow, inefficient, laborious, ineffective in the extraction of impurities, consumes a lot of energy, and requires many processes (Valasi et al. 2023). They often rely on large volumes of hazardous organic solvents, require long processing times, make complete removal of solvent residues challenging, and expose materials to high temperatures for extended periods, increasing the risk of thermal degradation (Easmin et al. 2015). The ideal extraction parameters were determined to be a 20‐min extraction duration, a leaf‐to‐water ratio of 200 g L^−1^, with a temperature of 75°C and grinding without agitation, can be used for extraction (López‐Carbón et al. 2019). A traditional extraction method is the extraction of phenolic compounds of the leaves of *Stevia rebaudiana* with water and ethanol solvents, with the extraction period up to 12 h (Ciulu et al. 2017). Hence, to overcome these limitations, several advanced extraction techniques have been developed.

Numerous unconventional extraction techniques have been employed, including Supercritical fluid extraction (SFE), pulsed electric field extraction (PEF), enzyme‐assisted extraction (EAE), ultrasound‐assisted extraction (UAE), pressurized liquid extraction (PLE), microwave‐assisted extraction (MAE), and subcritical water extraction (SWE) (Galanakis 2021). Over the last few years, various advanced separation methods have been devised with regard to the purification and enrichment of complex mixtures. The analysis and purification of steviol glycosides (SGs) have been done by high‐speed counter‐current chromatography (HSCCC), macroporous resin adsorption (MRA), ion exchange chromatography (IEC), high‐performance liquid chromatography (HPLC), and recrystallization. High‐voltage electrical processes, ultrasonic treatments, and microwave‐based techniques represent non‐traditional extraction approaches that can shorten processing time, operate at reduced temperatures, require less solvent, and deliver higher extraction efficiency with lower energy use compared to conventional methods (Kujundžić et al. 2017). Emerging extraction technologies are also valued for causing very little damage to the nutritional and functional qualities of the recovered compounds. This advantage aligns well with current innovation trends in the agri‐food and nutraceutical industries (Galanakis 2021). Modern techniques like microwave‐assisted extraction (MAE), ultrasound‐assisted extraction (UAE), supercritical fluid extraction, and pressurized hot water extraction (PHWE) have gained greater popularity compared to traditional approaches such as boiling, Soxhlet extraction, and refluxing (Kapadia et al. 2022). Different extraction methods for Steviol Glycosides (SGs) in Stevia were tabulated in Table 2.

**TABLE 2:** Different extraction methods for Steviol glycosides (SGs) in *Stevia*.

S. No	Extraction method	Instrument used	Extracted compounds	Study outcome	References
1	Ultrasound‐Assisted Extraction (UAE) in batch and continuous mode using water as green solvent	Ultrasonic generator (20 kHz, 64–144 W), batch with recirculation and continuous assembly	Steviol glycosides (mainly Stevioside and Rebaudioside A) + soluble solids	Ultrasound enhanced the degree of extraction by 9%–10% in comparison with traditional extraction; ultrasound tended to extract more stevioside; cleaner process and greater yield than the solvent‐based techniques.	Lima et al. (2021)
2	Ohmic Heating‐Assisted Water Extraction (OH‐AWE)	Ohmic heating system (electric field strengths 75, 150, 200 V/cm); moisture content adjusted to 20%–40%	Steviol glycosides (Stevioside, Rebaudioside A), phenolic acids (chlorogenic, syringic, caffeic, ferulic), total flavonoids & total phenolics	OH‐AWE gave higher stevioside (11.48–15.34 g/100 g DW), rebaudioside A (6.53–8.58 g/100 g DW), flavonoids, phenolics than water (UN‐W) and methanol (UN‐M) extractions. Optimal at 30% MC (200 V/cm) and 40% MC (150 V/cm). Suggested as potential green method for sweeteners rich in phytochemicals.	Moongngarm et al. (2022)
3	Subcritical Water Extraction (SWE) of *Stevia rebaudiana* leaves	Subcritical water extractor (230 bar, 100°C–150°C, 30–60 min, flow 2–6 mL/min), Box–Behnken statistical design	Steviol glycosides (Stevioside, Rebaudioside A), total phenolics, flavonoids, antioxidants; raffinate phase (chlorophylls, carotenoids, dietary fibers)	Optimum at 125°C, 45 min, 4 mL/min: yielded 38.67 mg/g stevioside & 35.68 mg/g rebaudioside A. Extract showed high phenolics (48.63 mg GAE/g), flavonoids (29.81 mg QE/g), DPPH scavenging (92.5%). Raffinate contained chlorophylls (31.91 mg/100 g), carotenoids (5.71 mg/100 g), and fibers (4.98%). Both extract & raffinate are valuable for sweeteners, fibers, and natural coloring agents.	Yildiz‐Ozturk et al. (2014)
4	Pressurized Liquid Extraction (PLE) of *Stevia rebaudiana* leaves	Pressurized liquid extractor (temperature up to 125°C, pressure, ethanol–water mixtures)	Steviol glycosides, total phenolics (TPC), flavonoids, antioxidants	Optimum at 125°C, 70% ethanol, 30 min: higher TPC & antioxidant activity vs. Soxhlet & UAE. Ethanol concentration favored extraction, while static time & pressure had little effect. Soxhlet yielded more steviol glycosides & flavonoids, but PLE gave cleaner extracts rich in phenolics & antioxidants.	Raspe et al. (2023)
5	Microwave‐Assisted Extraction (MAE) Optimized Using Response Surface Methodology (RSM) and Artificial Neural Network (ANN)	Microwave extraction system (40–200 W, ethanol–water solvent 0%–100%, extraction time 1–5 min); central composite design	Stevioside, Rebaudioside A, total extract	Optimum conditions: 4 min, 75% ethanol, 160 W, produced: 7.67% total extract, 19.58 mg/g stevioside, 15.3 mg/g Reb‐A: ANN model better predicted than RSM; RSM identified the effects of parameters.	Ameer, Bae, Jo, Lee, et al. (2017)

A wide range of analytical methods, such as liquid chromatography, capillary electrophoresis, nuclear magnetic resonance, and Fourier transform infrared spectroscopy, were also suggested for the characterization of stevia extracts. Among these, liquid chromatography‐mass spectrometry (LC–MS) and liquid chromatography in conjunction with ultraviolet‐photodiode array detection (LC‐DAD) were prominently used in recent times (Pacifico et al. 2017; Pavlíček and Tůma 2017). The use of ultrasound is highly efficient for extracting different types of compounds when compared with traditional methods such as maceration and thermal extraction. This efficiency is attributed to the mechanical action of ultrasound, which enhances solvent penetration into plant tissues and significantly improves the mass transfer of soluble compounds. The cavitational energy produced by ultrasound also disrupts plant cell walls, thereby promoting the release of intracellular contents into the solvent (Gasmalla et al. 2017; Kujundžić et al. 2017). Ultrasound has proven to extricate very well, even at short processing times. It appears not to influence the particle size distribution of the leaves of Stevia, but it appears to positively influence the extraction of soluble components (Lima et al. 2021). By optimizing and validating the UHPLC‐ESI‐MS/MS method, nine steviol glycoside derivatives were efficiently separated and quantified, that is, stevioside, Reb A, Reb B, Reb C, Reb D, Reb F, dulcoside A, rubusoside, and steviobioside was achieved within a short run time of about 7 min (Phungsiangdee et al. 2023).

To enhance recovery of bioactive constituents in the stevia leaves, methods including the microwave‐assisted extraction (MAE), pressurized liquid extraction (PLE), and pressurized hot water extraction (PHWE) are used (Ciulu et al. 2017; Németh and Jánosi 2019). Microwave‐assisted extraction (MAE) is a new extraction method that employs the electromagnetic properties of the microwaves to enhance the process (Panja 2018). Microwave‐assisted extraction (MAE) offers several benefits, including deep penetration and efficient extraction, resistance to contamination by impurities, and rapid processing. In recent years, it has been extensively used for isolating bioactive compounds from plants. SEM images of Stevia leaves subjected to ohmic treatment revealed significant disruption of cell membranes and adjacent cellular structures, leading to the release of intracellular compounds and increased extraction efficiency. The Ohmic heating‐assisted aqueous extraction was found to yield larger concentrations of stevioside, rebaudioside A, and overall flavonoid as well as increased antioxidant activity compared to untreated water extraction (UN‐W) and untreated methanolic extraction (UN‐M) (Moongngarm et al. 2022). Even though the extraction of leaves using Stevia has been reported to occur at relatively low levels of ultrasonic power and frequency (< 1200 W and less than 25 kHz), improper settings of these factors may negatively impact the extraction efficiency. This decrease is because too much energy can cause the production of free radicals due to oxidative pyrolysis of the cavitation bubbles, which can degrade the target compounds (Sivasankar et al. 2007). An ultrasound power of 100% (200 W) was reported to achieve the highest extraction efficiency of stevioside without causing any negative effects (Rouhani 2019). Raspe et al. (2023) studied the effect of ultrasound in the enhancement of compound extraction of the pre‐treated Stevia leaf and found that the ultrasound‐based extraction (UAE) using a reduced solvent‐to‐leaf ratio, lower temperature, and shorter extraction duration resulted in a greater yield of Reb A as compared to orbital agitation extraction.

### 4.2 Stevioside Traceability Using Advanced Analytical Techniques

Stevia is a non‐nutritive and low‐calorie sweetener that is commonly incorporated into a wide range of food and drink products. It provides a sweet taste without increasing the level of blood sugar, which is useful when a person is diagnosed with diabetes and wants to control the level of glucose in the body and prevent any related issues (Angelin et al. 2024; Pang et al. 2021). HPLC–UV, LC‐ MS/MS, high‐performance thin‐layer chromatography (HPTLC), two‐dimensional ultra‐HPLC, and near‐infrared spectroscopy (NIR) are some of the analytical methods used in the quantification of steviol glycoside. Among these, HPLC is the most common technique with a continuous requirement to improve its analysis processes (Cho et al. 2025). A study carried out on Soju aimed to determine the maximum permissible levels of steviol glycosides in food products and evaluate their daily intake. The developed separation and quantification techniques for steviol glycosides apply to the quality control of liquid foods, and the findings can serve as a basis for recommending safe daily Soju consumption to the public (Kim et al. 2024). Advanced analytical techniques for stevioside traceability are shown in Table 3. A validated HPLC‐UV method was developed for quantifying rebaudioside D in *Stevia rebaudiana* leaves (C18, 250 × 4.6 mm, 5 μm; 210 nm; acetonitrile/sodium phosphate buffer 32:68 v/v; flow 1 mL/min). The method showed good linearity (25–150 μg/mL, r^2^ ≥ 0.99), LOD 8.53 μg/mL, and LOQ 25.85 μg/mL. Recovery was 100% ± 10%, with precision RSD ≤ 2.79%. Rebaudioside D content was 0.43 and 0.46 g/100 g in Morita II and Criolla varieties, respectively, with no significant difference (*p* > 0.05) (Aranda‐González et al. 2014). The two *Stevia rebaudiana* cultivars cultivated in southeastern Mexico showed rebaudioside D levels between 0.43 and 0.46 g per 100 g of dried leaves, and the values were statistically comparable across both types (Aranda‐González et al. 2014).

**TABLE 3:** Advanced analytical techniques for stevioside traceability.

S. No	Study	Method used	Mobile phase & column	Instrumentation	Key findings	References
1	Waters JECFA Resolution Improvement	RPLC (LC‐UV, LC‐UV‐MS)	Columns screened: XSelect Premier HSS T3 (2.5 μm) & ACQUITY UPLC HSS T3 (1.8 μm); Mobile phase A: water:ACN (8:2) + 0.02% FA; B: ACN + 0.02% FA	Arc Premier LC with PDA detector (UV 210 nm, PDA 200–400 nm)	The method was improved by employing 2.5 μm particle‐size columns on the Arc Premier System to achieve a resolution of ≥ 1.5, and further enhanced to 2.0 using sub‐2 μm particle‐size columns. Optimization was carried out in line with ICH Q14 guidelines, and the resulting method is accurate, sensitive, reliable, robust, and enables more efficient separation for the analysis of commercial stevia extracts.	Yang et al. (2024)
2	UHPLC–ESI–MS/MS	UHPLC–ESI–MS/MS (Triple Quad, SRM mode)	Accucore RP‐MS C18 (100 × 2.1 mm, 2.6 μm); Mobile phase A: 0.05% FA in ACN; B: 0.05% FA in water; gradient 13 min	Thermo Ultimate 3000 UHPLC + TSQ Quantis triple quadrupole MS (ESI‐, SRM)	Calibration curves for steviol glycosides showed strong linearity (R^2^ = 0.9911–1.0000) across beverages, yoghurt, and snacks, with intra‐day precision below 15% RSD and recoveries within acceptable limits (70%–120%). Matrix‐matched calibration confirmed linearity (0.2–1.0 mg L^−1^) with recoveries mostly within 80%–120%. The method achieved low detection (LOD: 0.003–0.078 μg g^−1^) and quantitation limits (LOQ: 0.011–0.261 μg g^−1^), demonstrating suitability for detecting steviol glycosides in diverse food matrices.	Phungsiangdee et al. (2023)
3	UHPLC‐Orbitrap HRMS	UHPLC‐Orbitrap MS (HR, full‐scan + CID)	BEH Amide column (150 × 2.1 mm, 1.7 μm); Eluents: A = 0.05% formic acid in water, B = 0.05% formic acid in acetonitrile; gradient 13%–50%	UHPLC (Waters Acquity) + Orbitrap HR‐MS (Exactive, Thermo) with HESI‐II probe	A sub‐2 μm amide column enabled full separation of steviol glycosides within 30 min with high resolution. Optimal ionization was achieved in negative mode with 0.05% formic acid, yielding stable [M‐H]^−^ ions and reducing glucose loss. Precision was high (%RSD 2.1%–5.1%), recoveries exceeded 95%, and Rebaudioside A was the most abundant (23%–102%). Nine stevia extracts and one drink failed European regulation compliance, isosteviol was below detection, and steviol was detected in four samples (0.01%–0.03%).	Gardana and Simonetti (2018)
4	LC–QTOF MS/MS	LC–QTOF MS/MS (untargeted metabolomics)	Mediterranea Sea C18 (5 μm, 15 × 0.46 cm); Eluents: A = water +0.1% formic acid; B = methanol +0.1% formic acid; gradient 10%–100% B in 25 min	Agilent 1200 LC + QTOF 6540 UHD Accurate Mass MS; dual ESI (positive & negative)	LC–QTOF MS/MS profiling of *Stevia* leaves identified 89 compounds across polar and non‐polar extracts. Steviol glycosides and caffeoylquinic acids dominated the polar fraction, with several new steviol glycosides reported. High levels of caffeoylquinic acids, free amino acids, and fatty acid amides were also detected, alongside fatty acids, glycerolipids, purines, and a retinol derivative, highlighting the chemical diversity and bioactive potential of *Stevia*.	Molina‐Calle et al. (2017)
5	UHPLC–MS/MS (MRM)	HPLC–MS/MS profiling	Ascentis Express C18, 5 cm × 2.1 mm, 2.7 μm; Solvent A: 10 mM ammonium acetate, Solvent B: acetonitrile; Gradient elution, 8 min run	QTRAP 3200 (AB Sciex); Shimadzu LC‐20 ad, SIL‐HTc autosampler, ESI, MRM mode (neg. ion)	A rapid 8‐min UHPLC–MS/MS method was established for the separation and quantification of seven steviol glycosides—steviolbioside, stevioside, rebaudiosides A–C, rubusoside, and dulcoside—along with steviol and isosteviol. Utilizing negative‐mode ESI–MRM, the technique achieved very low detection limits: less than 1 ng/mL for steviol, 6 ng/mL for isosteviol, and below 15 ng/mL for the glycosides. Large‐scale analysis of more than 1100 stevia leaf extracts demonstrated substantial variability, with stevioside ranging from 2 to 125 mg/g, Reb A from 2.5 to 164 mg/g, Reb B from 0.5 to 50 mg/g, and Reb C from 1.5 to 125 mg/g, spanning roughly two orders of magnitude, showing independent variation across metabolites and demonstrating the method's strong utility for population‐scale analysis.	Shafii et al. (2012)
6	HPLC (UV detection)	HPLC (UV detection)	Agilent ZORBAX C18 (4.6 × 250 mm, 5 μm); isocratic: acetonitrile: 10 mM sodium phosphate buffer (pH 2.6) (32:68 v/v)	Agilent 1260 HPLC, UV detector (210 nm)	Simple and robust method for estimating stevioside and rebaudioside A in dairy and non‐dairy foods after Carrez/acetonitrile or aqueous extraction; LOD/LOQ for Reb A: 1.06–6.11/3.53–6.11 mg/kg; Stev: 1.68–9.71/5.60–9.71 mg/kg; recoveries ~99%–102%; RSD < 2.6%; suitable for regulatory QC and routine	Fayaz et al. (2018)

A fast and powerful isocratic HPLC technique was designed for the concentration of nine steviol glycosides in *Stevia rebaudiana* leaves in a Purospher STAR RP‐18 column at 50°C with 65% of water and 35% of acetonitrile. LOD of 0.0004 mg/mL, LOQ of 0.0038 mg/mL, excellent linearity at a maximum of 4.8 mg/mL (r^2^ = 0.9997 ± 0.0002), and 100.99 ± 2.01 percentage recovery were observed in the validation of Rebaudioside A. The precision was < 2% intra‐day and inter‐day, and the technique was not sensitive to small changes in operating conditions, so it was applicable in the laboratory and quality control (Bergs et al. 2012). HPLC remains the most straightforward and dependable method for analyzing steviol glycosides. The initial successful separation of stevioside and rebaudioside A using HPLC was reported by Hashimoto et al. (1978); a reversed‐phase HPLC method was developed to quantify stevioside and Reb A from *Stevia rebaudiana* , with water as the extraction solvent and solid‐phase extraction (SPE) for sample clean‐up, with separation achieved on a Luna HILIC column (acetonitrile/water, 85:15 v/v). The method showed a linear calibration range of 10–800 μg/mL, with recoveries of 99% ± 4.4% for stevioside and 100% ± 5.0% for rebaudioside A. Earlier, a hydrophilic packed column, Shodex OHpak M‐414, had been employed to detect only stevioside and rebaudioside A (Woelwer‐Rieck et al. 2010). Using an energy‐resolved, untargeted LC–MS/MS metabolomic approach, researchers detected 91 different diterpene glycoside compounds, including various SGs. Within this group, 16 SGs displayed previously unreported acetyl‐glycosyl modifications (Zhang, Li, et al. 2024; Zhang, Chen, et al. 2024).

## 5 Safety Assessment and Regulatory Aspects of Stevia

### 5.1 Food Safety

Stevioside is highly stable in saliva, gastric juice, and small‐intestinal conditions; human digestive enzymes do not hydrolyse it appreciably, so it passes essentially intact into the colon. In vitro digestion models (SGF/SIF assays) confirm that stevioside and its α‐1,6‐glucosylated derivatives reach the colon without significant degradation (Park et al. 2025). In the colon, stevioside is sequentially hydrolysed by gut bacteria (especially Bacteroides spp. and related anaerobes) first to steviolbioside and then to steviol, the aglycone backbone (Geuns et al. 2007). Liberated steviol is absorbed across the colonic epithelium and enters the portal circulation (Peng et al. 2026). In the liver, steviol undergoes extensive glucuronidation to form steviol glucuronide in urinary form (Wheeler et al. 2008). After oral stevioside, human and rodent studies show steviol glucuronide as the predominant circulating and excretory metabolite, with peak plasma levels reached within hours and elimination mainly via urine with some enterohepatic recirculation and fecal excretion in rodents (Geuns et al. 2007). Gut microbiota hydrolyses stevioside into steviol in the human colon, as the upper GI tract lacks enzymes for β‐glycosidic bond cleavage. Stevioside passes undigested to the colon, where Bacteroides species like *B. thetaiotaomicron* hydrolyse it stepwise to steviol, the aglycone absorbed via the portal vein. Steviol undergoes liver glucuronidation to steviol glucuronide, excreted renally in humans. This microbial step enables about 90% steviol recovery within 24–48 h (Kasti et al. 2022; Peng et al. 2026). In the colon, *Bacteroides* species produce enzymes that hydrolyse stevioside into its aglycone form, steviol, while other genera such as *Lactobacillus*, *Bifidobacterium*, *Clostridia*, and *Enterococci* play little or no role in this conversion. Stevia compounds can interact with the gut microbiome by exerting selective, strain‐dependent effects on bacterial growth, with most studies reporting either neutral or mild modulatory impacts rather than major dysbiosis. Stevia may affect microbial functions, including bacterial communication systems, and exhibit mild antimicrobial activity against specific pathogens without broadly disrupting beneficial microbes (Kasti et al. 2022).

For inclusion in food and beverage products within the European Union, food additives such as non‐nutritive sweeteners (NNS) must pass a stringent authorization process as specified in Regulation (EC) No. 1333/2008. Approval is granted only when scientific evidence demonstrates that the NNS is safe for consumption at the intended levels of use (Younes et al. 2019). Sweeteners are evaluated like any other food additive at the international level by the Joint FAO/WHO Expert Committee on Food Additives (JECFA), a body consisting of experts of the Food and Agriculture Organization (FAO) and the World Health Organization (WHO). The primary purpose of this committee is to reach values of health‐based guidance (HBGVs), such as the Acceptable Daily Intake (ADI) of every additive under assessment (Coro et al. 2025). Dietary exposure assessments indicate that high consumers, particularly toddlers, may slightly exceed the acceptable daily intake (ADI) of 4 mg kg^−1^ body weight per day, with intake estimates reaching approximately 4.3 mg kg^−1^ bw day^−1^ at the 95th percentile (European Food Safety Authority 2014). Findings highlight the importance of careful exposure assessment and clear risk communication, particularly for products frequently consumed by children, to ensure cumulative intake remains within safe limits.

### 5.2 Toxicological Evaluation

Clinical research has shown that steviol glycosides are safe to the human body, having neither acute nor subacute toxicity (Khilar et al. 2022). Crude extracts of Stevia can be more allergenic than stevia‐based sweeteners with 95% or higher steviol glycoside content since crude extracts of Stevia are more susceptible to retaining the allergenicity of steviol glycosides that are naturally found in the Asteraceae family (Urban et al. 2015), but this issue is not well studied yet. Research indicates that administering stevioside at doses of about 2000 mg per kilogram of body weight does not cause DNA aberrations or chromosomal abnormalities in mice, and that its coarse crystalline form does not exhibit mutagenic effects (Saeidnia and Abdollahi 2013). The Joint Expert Committee on Food Additives (2005), along with various international regulatory bodies for food safety, has determined that stevia, stevioside, and rebaudioside A are safe for use at their established intake levels, with no evidence indicating any genotoxic risk (Elnaga et al. 2016; Qiong et al. 2018). Additional evidence suggesting that Stevia is safe to consume is that it has not been associated with any side effects when used in large quantities in Japan, where populations have been taking Stevia in large quantities over the past few years (Singh and Rao 2005). In a recent study, 4 weeks of consuming a drink containing 25% of the allowable daily intake (ADI) of starch revealed no significant alterations in the gut microbiome, fecal fatty acids, or fasting cardiometabolic parameters, relative to products consumed by humans using 30 g of sucrose (Kwok et al. 2024). A majority of studies that have analyzed the significance of Stevia in human health have not found any side effects (Nikiforov et al. 2013; Uçar et al. 2018). In one experiment, Raman spectroscopy was used to analyze six commercial *Stevia* products, and it was discovered that three of them were counterfeit. These samples were found to contain sodium cyclamate along with small amounts of sodium saccharin (Jentzsch et al. 2016). Safety evaluations have reported no adverse findings for Reb M obtained through enzymatic bioconversion of Stevia leaf extract after purification, a process which employs UDP‐glucosyltransferase along with sucrose synthase enzymes, which are produced through genetically modified *K. phaffii* strains UGT‐a and UGT‐b, to be used as a food additive (Younes et al. 2019), for which regulations have to be carried out before being commercialized.

### 5.3 Global Regulatory Framework of Stevia

The Steviol glycosides (SGs) are widely used as a natural sweetener in food items. They were first used as sweetening agents in foods and drinks in Japan in the 1970s and 1980s (Mathur et al. 2017). South Korea, Malaysia, and Latin America, among other regions, slowly embraced the use of steviol glycosides as a sweetener. At present, they are allowed in 14 large food categories (including 53 sub‐categories), and the maximum permissible level is between 30 and 2500 mg/kg (Phungsiangdee et al. 2023).

The history of stevia safety can be traced in the tradition of brewing tea using the dried leaves of stevia, which has existed for over 1500 years among the Paraguayan (Ahmad et al. 2020). Increased awareness about the importance of consuming less sugar leads to an increase in the substitution of sucrose with Non‐nutritive sweeteners (NNS) and no‐calorie sweeteners in food and beverages (Phonsuk et al. 2021). There has been a growing shift in the food sector towards natural sources of Non‐nutritive sweeteners (NNS), including tagatose, thaumatin, and, most recently, the steviol glycosides (Phonsuk et al. 2021). These sweet compounds have been authorized as natural sweeteners in more than 60 countries and are recognized by major regulatory bodies, including the FAO/WHO Joint Expert Committee on Food Additives (JECFA), the European Food Safety Authority (EFSA), and the U.S. FDA. Currently, over 30 different SGs have been identified in Stevia, such as stevioside, steviolbioside, several rebaudiosides (A, B, C, D, E, F), and dulcoside, highlighting the plant's rich diversity of naturally occurring sweet compounds (Schiatti‐Sisó et al. 2023). E 960, Steviol glycosides, are legal food additives in the European Union, and may be used in all classes of foods with certain manufacturing and quality standards defined. Stevia sweeteners are legal in numerous regions, such as Japan, Brazil, the European Union, China, and the United States, among others (Prakash et al. 2008, 2014). The FDA has recognized highly purified steviol glycosides as Generally Recognized as Safe (GRAS), and hence they are permitted to be used in food products (Perrier et al. 2018). Stability Testing for steviol glycosides is based on the regulatory views of safety and quality globally.

The Joint FAO/WHO Expert Committee has set down the safety guidelines that steviol glycosides need to have a minimum purity of 95%. In addition, favorable regulatory frameworks have helped to boost the growing stevia market, with several GRAS notifications passed to be used in a variety of food products (Perrier et al. 2018). EFSA reviewed the safety of steviol glycosides as food additives in 2010 (Exposure Assessment EFSA 2011). Following the EFSA assessment in 2015 (EFSA 2015; Younes et al. 2018), Reb M was added to the food additive steviol glycoside (E 960) in Commission Regulation (EU) No. 231/2012. GRAS Notice No. 375 of the stevia associated with glycosylated enzyme‐treated stated zero evidence of genotoxic activity in both in vitro and in vivo studies (Younes et al. 2018). Moreover, the risk of using LNCSs more and possibly surpassing the acceptable daily intake (ADI) is also discussed because of the unavailable detailed information on the product labelling concerning their contents (Barraj, Bi, and Tran 2021; Barraj, Scrafford, et al. 2021; Coyle et al. 2021). Definition of steviol glycosides (E 960a–d) as outlined in Commission Regulation (EU) No. 231/2012 was tabulated in Table 4.

**TABLE 4:** Description of steviol glycosides (E 960a–d) as defined in Commission Regulation (EU) No 231/2012 (Castle et al. 2024).

E number	Food additive
E 960a	Steviol glycosides from stevia
E 960c (i)	Rebaudioside M produced via enzyme modification of steviol glycosides from stevia
E 960c (ii)	Rebaudioside M produced via enzymatic conversion of highly purified rebaudioside A stevia leaf extracts
E 960c (iii)	Rebaudioside D produced via enzymatic conversion of highly purified rebaudioside A stevia leaf extracts
E 960c (iv)	Rebaudioside AM produced via enzymatic conversion of highly purified stevioside stevia leaf extracts
E 960d	Glucosylated steviol glycosides

### 5.4 Acceptable Daily Intake

Steviol glycosides (E 960) are naturally sweet substances whose application as sweeteners is controlled by the European Parliament and the Food Additives Council regulation (EC) No. 1333/2008. The Scientific Committee on Food (SCF) conducted evaluations of stevioside in 1984, 1988, and 1999. During the period from 2000 to 2009, the Joint FAO/WHO Expert Committee on Food Additives (JECFA) assessed steviol glycosides (E 960) multiple times and confirmed their safety. Subsequently, the EFSA Panel on Food Additives and Nutrient Sources Added to Food (ANS) studied these compounds in 2010 and formulated an Acceptable Daily Intake (ADI) of 4 mg per kilogram of body weight per day. Further adjustments to the estimated intake were carried out by EFSA in 2011 and again in 2014, following a request to extend their permitted use (EFSA 2010, 2015; European Food Safety Authority 2014; Exposure Assessment EFSA 2011; WHO 2007, 2009; WHO TECHNICAL REPORT 2012). Oral intake of steviol glycosides at doses of about 1880 mg/kg over four weeks caused increases in oxidative stress and the frequency of chromosomal abnormalities in rats. Nevertheless, in vivo experiments with doses as high as 8000 mg/kg did not reveal any genotoxic effects of steviol (Chatsudthipong and Muanprasat 2009; Yılmaz et al. 2022).

The absorption of Stevia is influenced by the molecular weight of its compounds, with the steviol content corresponding to 33% of the weight of rebaudioside A (Reb A) and 40% of stevioside (STV). The FAO/WHO Joint Expert Committee on Food Additives (JECFA) has set the acceptable daily intake for Stevia, calculated as steviol equivalents, at 0–4 mg per kg of body weight per day (Gupta et al. 2016). SGs, particularly STV and Reb A are considered safe for inclusion in dietary supplement formulations according to the U.S. FDA. The permitted daily intake has been set at 7.9 mg per kg of body weight per day for humans, and 25 mg/kg body weight/day in rats (Exposure Assessment EFSA 2011; Regulation (EU) 1169/2011 (EU 2011) 2011). In Thailand, the national FDA permits the use of steviol glycosides under Ministry of Public Health Notification No. 444 B.E. 2566 (2023), allowing their incorporation into 14 approved food and beverage categories. Both JECFA and EFSA set the acceptable intake limit at 4 mg/kg body weight per day, expressed as steviol equivalents (European Food Safety Authority 2014). The EFSA Panel on Food Additives and Nutrient Sources Added to Food (ANS) evaluated consumer exposure to steviol glycosides using the highest allowable usage levels as the basis for their assessment (Kujur et al. 2010). The European Food Safety Authority (EFSA 2015) listed the maximum allowed percentages of steviol glycosides, where the permitted level in non‐alcoholic beverages generally ranges from 350 to 600 mg/L, while in desserts and similar products it ranges from 110 to 600 mg/kg, with certain specialized products allowing levels up to 10,000 mg/kg. EFSA also revised the maximum permitted level for steviol glycosides in instant coffee, tea, and instant cappuccino to 29 mg/L (as steviol equivalents), replacing the earlier level of 10 mg/L reported in the 2014 assessment (EFSA 2015). Results Data derived from conservative intake calculations for SGs in both adult and pediatric groups show that intake levels may exceed the established ADI when calculated based on the maximum proposed use levels (Tanaviyutpakdee et al. 2025).

## 6 Applications in Functional Foods and Health‐Oriented Product Development

Increasing safety concerns associated with artificial sweeteners have contributed to a shift in consumer preference towards alternative sweetening agents. Steviol glycosides (SGs) are produced from stevia leaves and serve as natural ingredients in many food production processes. Since stevia grows widely around the world, there is a constant and plentiful supply of fresh leaves, promoting the SGs in a low‐cost supply (Chuo et al. 2022). Steviol glycoside products are known to be natural, calorie‐free, and safe. They are non‐teratogenic, non‐carcinogenic, non‐mutagenic, subacute or polar toxicity‐free (Abbas Momtazi‐Borojeni et al. 2017), Consumers greatly favor non‐nutritive sweeteners worldwide. Natural sugars naturally contain calories and those looking for a sweet taste with little or no calories will have to procure alternate sources (Prakash et al. 2014). Non‐nutritive sweeteners are not easy to develop because the taste characteristics of sucrose are different from those of non‐caloric sweeteners in various ways, including sweetness duration, intensity, flavor, taste perception, and adaptive response (Prakash et al. 2008). Artificial sweeteners (ASs) are promising sugar alternatives because of their low‐calorie content and negligible effect on blood glucose levels. Sweeteners, including stevia, sucralose, and aspartame, replicate the sweetness of white sugar without inducing hyperglycemia, making it suitable for individuals with diabetes (Begum et al. 2025). Food products developed and the application of Stevia in various food products are shown in Table 5. Since 1994, stevia has been accepted and used in Egypt as a safe, natural sweetener in herbal teas, dairy products, baked goods, and soft drinks (Ghonema 2014).

**TABLE 5:** Food products developed and the application of Stevia.

S. No	Food product	Application	Ingredients (key formulation)	Key findings	References
1	Okra mucus jelly drink	Anti‐diabetic functional beverage	Okra mucus (mucilage), carrageenan (0.10%–0.30%), low‐calorie sugars (sucralose/stevia 0.02%–0.04%)	Best formula (0.3% carrageenan +0.04% stevia) showed good viscosity (279.5 cps), gel strength (4.53 N), phenolic content (76.63 mg TAE/g), fiber (1.29 g/100 g), and anti‐diabetic activity (IC_50_ = 32.60 mg/mL); texture was favored, while taste, aroma, and color had moderate acceptance.	Jariyah et al. (2021)
2	Probiotic milk gels enriched with red beetroot	Therapeutic dairy product (probiotic + functional)	Milk gels, *Lactobacillus acidophilus* , red beetroot bioactive compounds (phenolics, anthocyanins), steviol glycosides (sugar substitute)	Maintained probiotic viability (> 9 log cfu/mL); stevia and beetroot bioactives supported the growth of *L. acidophilus* ; enhanced antioxidant activity (DPPH, FRAP), texture, color, and sensory acceptance; positive synergistic effect for probiotic delivery.	Ozdemir and Ozcan (2020)
3	Jelly candies with rosemary extract & stevia	Antioxidant‐enriched confectionery	Fructooligosaccharides, inulin, stevia, aqueous rosemary extracts (RE74: 73.9 mg polyphenols/g; RE146: 145.6 mg polyphenols/g)	Addition of 0.26 g RE146/kg increased polyphenol content (197 → 411 mg GAE/g) and antioxidant capacity (1.77 → 4.14 μmol Trolox/g) without altering pH, °Brix, texture, color, or sensory acceptance; rosemary polyphenols were stable to cooking and improved oxidative stability.	Cedeño‐Pinos et al. (2020)
4	Ice cream with stevia	Diabetic‐friendly dessert	Formulated ice cream (FIC) and 5 commercial brands; sucrose replaced with stevia	Replacement of sucrose with stevia reduced total solids, fat, ash, freezing point, and hardness, but increased protein, viscosity, and sensory scores; FIC had the highest sensory 5acceptability; suitable for diabetic and general consumers.	Ahmed et al. (2023)
5	Low‐calorie muffins with stevia	Diabetic‐friendly/weight management bakery	Muffins with dried stevia leaf powder replacing sucrose at 25%, 50%, 75%, 100%	Stevia substitution increased protein, fiber, minerals (K, Ca, Mg, P), total phenols, flavonoids, and antioxidant activity (DPPH); affected physicochemical properties (diameter, thickness, spread, firmness, springiness, color) and organoleptic attributes; 25:75% (stevia: sucrose) muffins had the highest sensory acceptability.	Ahmad and Ahmad (2018b)
6	Sugar‐free chewing gum with stevia	Natural sweetener alternative to aspartame	Sorbitol, xylitol, stevia, and aspartame (for comparison)	Stevia can replace aspartame without affecting color, texture, or sensory properties; xylitol substitution reduced hardness and sensory acceptability; stevia is suitable for natural sugar‐free chewing gum production.	Aykut and Kirkin (2025)
7	Functional gummy candies with stevia & pistachio extract	Antioxidant‐enriched reduced‐sugar confectionery	Gelatin, starch, pistachio green hull extract (1%–5%), stevia (0.013%–0.040%)	Optimized formulations provided reduced‐sugar product (50% reduction, 12% sucrose), high total phenolic content (680.31 mg GAE/100 g), strong antioxidant activity (IC_50_ = 277 μg/mL), stable texture and storage; eliminated need for artificial flavors and synthetic colorants.	Roudbari et al. (2024)
8	Oatmeal cookies with stevia	Functional/dietotherapeutic cookies	Oatmeal, stevia aqueous extract (25%–100% replacement of sucrose)	Stevia substitution improved nutritional (protein, fiber, bioactive compounds) and biological properties (ACE inhibition, anti‐diabetic, antioxidant); sensorial and physicochemical properties were acceptable; functional cookies could help prevent metabolic syndrome.	Góngora Salazar et al. (2018)
9	Chocolate flavored milk with stevia & monk fruit	Sugar‐reduced functional beverage	Chocolate milk, stevia (5–100 ppm), monk fruit extract (50–100 ppm)	Optimized combination (56.27 ppm stevia +81.9 ppm monk fruit) achieved 50% sugar reduction, masked stevia bitterness and metallic aftertaste, improved overall sensory attributes (overall liking 6.78/9, sweetness 6.47/9), enhancing acceptability compared with control.	Mahato et al. (2021)

Stevia showed a slight improvement in the glycemic response, whereas sucrose and sucralose were shown to reduce glycemic response and insulin sensitivity when used in food products. Nonetheless, ongoing evidence is still not enough to conclusively recommend the widespread use of artificial sweeteners (Villaño et al. 2021). Many beverage companies have begun the addition of steviol glycosides (SGs) as sweeteners in their products. For example, the world's leading fruit juice distributor Coca‐Cola has been using Rebaudioside A (Reb A) in its beverages and has introduced Coca‐Cola Life (with stevia), which helped them achieve calorie reduction while maintaining the sweetness (Lemus‐Mondaca et al. 2012). When stevia is used as a sugar substitute in muffins, the product can be classified as a functional muffin because of its associated health benefits. This study supports the development of low‐calorie muffins formulated with stevia, which can enhance their nutritional value and help in the prevention of various health conditions such as obesity and diabetes (Ahmad and Ahmad 2018b). Rebaudioside A (Reb A) is considered the primary sweetener when functional sugar‐free yoghurt is designed for diabetic and obese patients. Ice creams made with Rebaudioside D (Reb D) and Rebaudioside M (Reb M) exhibit a sweeter, more pleasant, creamier, and milkier aftertaste compared to those made with Reb A, which remains the most commercially used steviol glycoside in food products (Muenprasitivej et al. 2022). In Brazil, biscuits most frequently contain steviol glycosides as high‐intensity sweeteners (HIS), and consuming baked goods formulated with these sweeteners has been shown to be toxicologically safe (Nicoluci et al. 2024).

The diet containing stevia‐based sweetener, a well‐balanced diet, and moderate exercise resulted in a decrease in body weight, waist circumference, and BMI by the end of 90 days. This sweetener reduced daily caloric intake by 90 kcal, and they estimate that this amounts to a monthly caloric deficit of 2700 kcal. Such an effect may account for the improvement in anthropometric parameters without any changes in metabolic parameters (Raghavan et al. 2023). Consumer demand for sugar‐free and natural products is rising as sugar consumption has been associated with multiple health problems, such as diabetes, dental disorders, cardiovascular diseases, and cancer. Consequently, the food industry has increasingly focused on developing products that use sweeteners as sugar substitutes (Ashwath Kumar and Sudha 2021). Artificial sweeteners have been associated with some health concerns; it is therefore recommended that the consumption of these be moderated (de Diniz et al. 2023). Chewing gums that contained stevia and xylitol were found to be effective in inhibiting the growth of *Streptococcus mutans* bacteria in saliva (Shinde and Winnier 2020).

## 7 Consumer Acceptance and Labelling Trends

The growing awareness of consumers and their desire to cut down the amount of sugar in their diets has contributed to the popularity of food products that are developed, and hence sugar can be substituted with non‐nutritive sweetening agents (Kołodziejczyk and Nowak 2025). Food manufacturers are reducing the usage of sugar in products or substituting it with low and no‐calorie sweeteners in response to evolving consumer expectations and regulatory government policies (Barraj, Bi, and Tran 2021). Steviol is generally 60%–70% of the total glycosides, and has therapeutic value, because of its ability to induce pancreatic insulin secretion that helps regulate diabetic conditions and other metabolic abnormalities. Although it has a sweet taste, the compound has a faint pungency and aftertaste, which can also reduce its overall palatability (Yadav et al. 2011; Yılmaz et al. 2021). Stevioside and Reb A are the most important sweeteners of SGs, and the commercially available in the market, as per the research conducted by the GNPD (Mintel Global New Products Database). Both compounds have a slight bitter and liquorice‐like aftertaste that presents problems in product formulation (de Medeiros et al. 2019; Gwak et al. 2012). The tastes of Rebaudioside D (Reb D) and Rebaudioside M (Reb M) in the mouth were comparable to those of sucrose, whereas Rebaudioside A (Reb A) exhibited noticeable bitterness. Compared to sucrose, Reb D and Reb M generated a prolonged sweet taste sensation (Tao and Cho 2020). Several studies have shown that the minor SGs, Reb D and Reb M, deliver a sweeter perception with much less bitterness than Rebaudioside A (Reb A) and perform well in a variety of products without affecting the quality of flavor (Hellfritsch et al. 2012). Prakash et al. (2014) suggests that Reb M demonstrates decreased bitterness and astringency in comparison with Reb A.

Rebaudioside M and Rebaudioside D showed a rapid onset of sweetness, a short aftertaste, and a negligible bitterness. Rubusoside and Stevioside, on the other hand, induced a sharp and instant bitter taste with an aftertaste. Steviol glycosides with larger substitutions at position C‐19 or more substitutions in the C‐19 position better endure the desorption process, and therefore the sweetness attenuation potential is faster. On the other hand, the components whose number of glucosyl groups was less, for example, Rubusoside and Stevioside, were desorbed to a lower degree and thus exhibited a stronger bitter aftertaste (Tian et al. 2022). A revised EU labelling regulation that took effect in August 2021 reclassified E960 as “steviol glycosides from Stevia” (E960a) to better communicate the ingredient's natural plant origin. Although consumer awareness of steviol glycosides remains limited, this update aims to promote greater transparency and trust through clearer labelling (Cargill 2021). Rebaudioside D and Rebaudioside M are found in trace quantities in the leaves of the stevia plant, but have a sweetness spectrum similar to sucrose with a cleaner and more natural taste compared to the primary glycosides. However, conventional methods of extraction for these compounds from plants are expensive and fall short because of the increasing demand on the market. As a consequence, the manufacturing methods have changed to rely on microbial conversion and enzymatic synthesis.

Contaminants found in stevia include mycotoxins, nicotine, and other microorganisms, and heavy metals like mercury, cadmium, and lead. Pyrrolizidine alkaloids (PAs) are common in stevia plants, and suppliers should be careful to monitor their levels. Furthermore, the product needs to be closely monitored for glyphosate residues and polycyclic aromatic hydrocarbons (PAHs) to help ensure product safety. Regulation (EU) 1169/2011 (EU 2011) (2011) outlines the requirements of providing information about food to consumers including the product containing sweeteners such as stevia extract. It requires indications on the labels of foods that contain additives to indicate what category the additive falls into (in stevia “sweetener”), the specific name of the additive or its E number (E960a or E960c). The name “steviol glycosides (E960)” has been changed to “steviol glycosides from Stevia (E960a).” A transitional period has been introduced to enable manufacturers to implement the new labelling from 2023. Companies, including Cargill, are promoting engagement among their European customers to make ingredient labelling updates as soon as possible, as clearer labelling that highlights the plant origin of stevia is likely to improve consumer transparency and positively impact stevia demand.

## 8 Conclusion and Future Prospects

Stevia glycosides are natural, low‐caloric, and non‐toxic compounds possessing multiple functional characteristics, and thus, they are highly adaptable in the sweetener industry. They show possible pharmacological activities such as antihypertensive and hypoglycemic, antidiabetic, anti‐inflammatory, antibacterial, and antitumor, although their exact mechanism still needs to be clarified. Stevia glycosides have a wide range of uses in the healthcare, agriculture, livestock management, and food sector. This review highlights the use of steviol glycosides as non‐calorie sweeteners, nanostevia formulations, biological activities, recent advances in extraction from conventional to non‐conventional methods for enhanced stevioside extraction for food safety and toxicological studies, the global regulatory framework as per different countries and their traditional history, purity requirement standards, health‐oriented product development, and future perspectives such as their use in the medical field, production of enhanced products, and fortified products whose limits of usage still need to be studied. This review paper serves as a kickstart for promoting stevia to the world for a better future. Further research should emphasize green extraction technologies, improved sensory properties through enzymatic modification, advanced analytical traceability, comprehensive clinical evaluation, and regulatory harmonization.

## Author Contributions

**Aswin Sakthivel Manoharan:** conceptualization, investigation, writing – original draft, methodology, visualization, writing – review and editing, software, formal analysis, data curation, resources. **Ramesh Kumar Selvan:** investigation, funding acquisition, validation, visualization, formal analysis, project administration, supervision, data curation.

## Funding

The authors have nothing to report.

## Ethics Statement

This research is a literature‐based study that does not require any ethical documentation regarding the use of animal testing or human subjects.

## Conflicts of Interest

The authors declare no conflicts of interest.

## Data Availability

Data sharing not applicable to this article as no datasets were generated or analysed during the current study.
